# Tuning the Properties of Polyvinylidene Fluoride/Alkali Lignin Membranes to Develop a Biocatalytic Membrane Reactor for an Organophosphorus Pesticide Degradation

**DOI:** 10.3390/membranes14090186

**Published:** 2024-08-28

**Authors:** Serena Regina, Giuseppe Vitola, Rosalinda Mazzei, Lidietta Giorno

**Affiliations:** National Research Council of Italy, Institute on Membrane Technology, CNR-ITM, 87036 Rende, Italy; s.regina@itm.cnr.it (S.R.); g.vitola@itm.cnr.it (G.V.); r.mazzei@itm.cnr.it (R.M.)

**Keywords:** bioremediation, alkali lignin, polyvinylidene fluoride, organophosphorus pesticides, biocatalytic membrane reactor, enzyme immobilization, blended membrane

## Abstract

It has been observed that the immobilization of a phosphotriesterase enzyme (PTE) onto polyvinylidene fluoride (PVDF) membranes significantly decreased the enzyme activity, and this negative effect was attributed to the hydrophobic character of the membrane. The indirect indication of this reason was that the same enzyme immobilized on other membrane materials bearing hydrophilic character showed better performance. In this work, we provide direct evidence of the mechanism by immobilizing a PTE on a PVDF membrane hydrophilized by blending it with alkali lignin (AL). The PTE was immobilized on PVDF membrane by a covalent bond with the same procedure used in earlier studies to attribute changes in enzyme activity solely to the wettability properties (and not to the material chemistry). The activity of the PTE immobilized on the PVDF membrane hydrophilized with AL was 50% higher than that of the enzyme immobilized on the PVDF hydrophobic membrane. Further improvements of the membrane structure tailored for the development of a biocatalytic membrane reactor (BMR) were also promoted. In particular, the performance of the BMR was studied as a function of the thickness of the membrane, which allowed us to modulate the residence time into the enzyme-loaded membrane pores while maintaining the flow rate through the pores at a constant.

## 1. Introduction

Biocatalytic membrane reactors (BMRs) combine the unmatched specificity and efficiency of enzymes with mass transport properties through membranes and represent a suitable technology for production, processing, and analysis [1]. The current trend towards green technologies makes BMRs very attractive because they (i) do not require additives, (ii) can operate at moderate temperature and pressure, and (iii) reduce the formation of by-products and enhance the conversion of product-inhibited reactions. To date, most published works on the development of BMRs have focused on enzyme immobilization techniques by using porous/mesoporous membranes originally designed for other applications, while studies addressing the preparation of membranes with specific properties for use in BMRs are still lacking [2,3,4,5]. General requirements for a biocatalytic membrane are uniform enzyme loading, high activity/selectivity, and high stability as well as constant transport/separation performance. The materials chemistry, physical–chemical properties, configuration, membrane structure, and topography at the nanoscale play a crucial role in influencing these requirements, and their control is essential to addressing engineering constraints for specific applications.

Phosphotriesterases are enzymes that can hydrolyze phosphoester bonds in organophosphates (OPs) [6,7], a class of toxic compounds used in the production of pesticides, among other things, and represent an environmentally friendly strategy for the decontamination of OPs [8].

PVDF is a polymer commonly used for the preparation of membranes employed in water treatment because it is easily processable and forms membranes with high chemical and thermal stability, high mechanical strength, and well-controlled porosity [9]. The use of PVDF for the immobilization of enzymes is limited by its hydrophobic nature, which hinders the interaction of the biocatalyst with water-soluble substrates, resulting in low catalytic activity [10,11]. Enzyme activity usually increases with water activity; in particular, hydrolytic enzymes like phosphotriesterases have demonstrated better performance when immobilized on hydrophilic membranes [11,12,13]. The main strategies to hydrophilize PVDF membranes are the coating, grafting, and blending modification methods [14]. Blending is a well-scalable one-step process and overcomes the problem of the detachment of the surface layer by unstable coating. Nevertheless, the poor compatibility of hydrophilic polymers with the hydrophobic matrix of PVDF has been a problem. Alkali lignin (AL) is a biopolymer derived from vegetable sources [15]. Lignin has been used as a surface modifier [16] and more recently, either as a blending additive in the polymer solution [17] or as a natural green polymer for membrane fabrication by using a deep eutectic solvent [18]. The good miscibility with the hydrophobic matrix of PVDF is due to the hydrophobic/hydrophilic nature of AL, consisting of phenyl and hydroxyl groups. From the mixing between AL and PVDF in a non-toxic solvent (dimethyl sulfoxide, DMSO), a high membrane hydrophilization can be obtained. This work aimed to hydrophilize PVDF membranes, use them as support for the immobilization, and compare their performance in the degradation of paraoxon pesticide with that of PTE loaded on PVDF hydrophobic membrane. Using the same protocol, both hydrophobic PVDF and hydrophilic PVDF/AL membranes were functionalized to covalently bind PTE to the PVDF by applying the amination method described in our previous works [19]. This would better prove that the observed decrease in activity of PTE immobilized on the PVDF membrane is due to the hydrophobic properties of the membrane. Although evidence of the negative influence of hydrophobicity on the activity of immobilized PTE has been described in the literature [11], the behavior was observed when different base materials (such as regenerated cellulose, nylon, polyether sulfone) were used and it remained unclear whether the chemistry of the material played an important role in addition to wettability. The results showed that the specific activity of the enzyme decreased significantly after immobilization and that the enzyme immobilized on PVDF/AL showed better performance compared to the enzyme immobilized on a pure PVDF membrane. PTE was then immobilized on PVDF/AL prepared with different mass ratios. For the mass ratio leading to better performance, the membrane thickness and the associated void volume, which represents the reactor volume, were the main parameters investigated to increase the residence time and its influence on pesticide degradation.

## 2. Materials and Methods

### 2.1. Materials

Polyvinylidene fluoride (PVDF, Solef^®^ 6010) was supplied by Solvay Specialty Polymers (Bollate, MI, Italy). Alkali lignin (AL; MW: 11,646 g/mol; density: 1.3 g/cm^3^ at 25 °C) with a low sulfonate content was purchased from Sigma Aldrich (Milan, Italy). HEPES, trizma, phosphate-buffer solution (PBS), a bicinchoninic acid (BCA) protein assay kit, glutaraldehyde (GA), 1,5-diamino-2-methylpentane (DAMP), paraoxon-ethyl, ninhydrin, ethanol, and concentrated sulfuric acid (H_2_SO_4_, 96% purity) were purchased from Merck (Darmstadt, Germany). Dimethyl sulfoxide (DMSO, anhydrous ≥99.9%) was purchased from VWR Chemicals (Milan, Italy). The phosphotriesterase (PTE) enzyme was from Sulfolobus sulfataricus (Detoxizymes srl, Italy) [20]. 

### 2.2. Membrane Preparation and Functionalization

The PVDF and PVDF/AL membranes used in this work were prepared and functionalized as briefly described below [17,19].

#### 2.2.1. Membrane Preparation

The membrane casting solution was prepared by dissolving PVDF (20 wt.%) in DMSO (80 wt.%) and stirring it at 60 °C for 1 h. The PVDF solution was degassed at room temperature (22 ± 1 °C) for 24 h, and then poured onto a glass plate using a casting knife (Elcometer) with a gap of 250 μm. The PVDF wet film was immersed in deionized water (non-solvent) at room temperature (22 ± 1 °C) for 30 min. The dope solution/water bath ratio was 1:50, respectively. Every 4 h, 3 times, the water bath was replaced with fresh deionized water. 

The PVDF/AL solutions were prepared by first dissolving the PVDF in DMSO at 60 °C for 1 h, and after the degassing of the solution, AL along with sulfuric acid (1 wt.%) were added and mixed for 5 h at 130 °C. The PVDF/AL solution was degassed at room temperature (22 ± 1 °C) for 24 h, and then poured onto a glass plate using a casting knife (Elcometer) with different gaps. The percentages of AL mass used in the dope solution were 10% (PVDF_90_/AL_10_), 25% (PVDF_75_/AL_25_), and 50% (PVDF_50_/AL_50_). In order to study the influence of the void volume on the degradation of paraoxon-ethyl, membranes with different thicknesses were also prepared. To do this, the gap of the casting knife was set at 250, 550, and 885 μm.

#### 2.2.2. Membrane Chemical Functionalization and Enzyme Immobilization

Membranes were cut into disks with an area of 17.34 cm^2^ and were thoroughly washed with ultrapure water. The functionalization of the membranes for the covalent attachment of phosphotriesterase was carried out by using the method reported in [19]. In particular, the PVDF or the PVDF/AL membranes were soaked in a 1,5-diamino-2-methylpentane solution (DAMP, 2M) in carbonate buffer pH 11 for 3 h at 50 °C to introduce amino groups onto the surface of the membranes (PVDF-DAMP, PVDF/AL-DAMP). The amino-functionalized membranes were then reacted with a 10% (*v*/*v*) glutaraldehyde solution at 25 °C for 2 h to graft reactive aldehyde groups by exploiting the Schiff base formation mechanism (PVDF-DAMP-GA or PVDF/AL-DAMP-GA). Afterward, a solution of phosphotriesterase (13 mL, 0.1 mg·mL^−1^) prepared using HEPES buffer 20 mM (pH 8.5) was incubated with the different aldehyde-functionalized membranes at 25 °C for 2 h under gentle stirring to prepare the biohybrid membranes. The rinsing with the same buffers was carried out to eliminate non-covalently bound enzymes; after that, the biocatalytic membranes were stored at 4 °C. The amounts of bounded enzyme were calculated by mass balance between the initial, final, and washing solutions according to Equation (1):

C_i_ V_i_ = C_f_ V_f_ + ∑C_ws_ V_ws_ + m (1)

where m is the mass (mg) of the immobilized enzyme, and C and V are the concentration (mg⋅mL^−1^) and volume (mL), respectively; the subscripts i, f, and ws refer to the initial, final, and washing solutions, respectively. The enzyme concentrations were evaluated by using the BCA assay kit based on a standard curve constructed by using bovine serum albumin protein. The correlation coefficient between the absorbance and BSA concentration was 1.01 mL⋅mg^−1^⋅cm^−1^. The amount of immobilized enzyme (mg) was normalized for the membrane weight (g).

### 2.3. Membrane Characterization

The PVDF and PVDF/AL membranes containing increasing amounts of AL (PVDF_90_/AL_10_, PVDF_75_/AL_25_, and PVDF_50_/AL_50_) were characterized by means of a scanning electron microscope (SEM), attenuated total reflectance–Fourier transform infrared (ATR-FTIR) spectroscopy, pore size, static water contact angle, and surface zeta potential measurements.

Zeiss-EVO MA10 SEM and Phenom Pro X desktop SEM (Phenom-World, Eindhoven, The Netherlands) were used to study the membrane structure. Membranes were frozen and fractured in liquid nitrogen. A thin conductive gold layer was deposited (Quorum Q150 RS) on membranes in order to assure imaging resolution and to prevent electrical charging. Images were taken at 350× (for the cross-sections) and 5000× (for the top views) magnification with an accelerating voltage of 15 kV.

ATR-FTIR spectroscopy (UATR crystal Diamond/ZnSe-Spectrum One System by PerkinElmer Italia (Milan, Italy)) was employed to investigate the grafting of functional groups on the membranes as well as the presence of the immobilized phosphotriesterase. Spectra were recorded in the wavenumber range 4000.00–650.00 cm^−1^ at a resolution of 4 cm^−1^. 

Pore size measurements were carried out by employing the porometer instrument (POROLUX^TM^ 1000 SERIES, Alfatest, Milan, Italy) based on the pressure step/stability method. The membrane samples (membrane area: 3 cm^2^) were wetted with Porefill 125 (surface tension: 16.39 ± 0.02 mN m^−1^) for 30 min prior to testing (porometer temperature: 21.0 °C).

Porosity (ε) was calculated by applying the following Equation (2) [21]:

(2) $$\varepsilon \;\left(\%\right)=\frac{\left(\frac{\left(wtw-wtd\right)}{\rho s}\right)}{\left(\frac{\left(wtw-wtd\right)}{\rho s}+\frac{wtd}{\rho p}\right)}\times 100$$

where *wt_w_* is the membrane wet weight, *wt_d_* is the membrane dry weight, ρ_s_ is the solvent density (ethanol: 0.789 g/cm^3^; deionized water: 1 g/cm^3^), and ρ_p_ is the polymer density (PVDF6010: 1.75–1.8 g/cm^3^; AL: 1.3 g/cm^3^) [22,23]. The polymer density for the PVDF/AL membrane was calculated by considering the weighted average of the mass fraction densities [24]. For porosity calculation, three pieces of the same membrane were weighed before and after the immersion in solvent for 15 min.

Colorimetric tests were performed using a UV-Vis spectrophotometer Perkin-Elmer Lambda EZ. The ninhydrin test was carried out by dipping a piece of membrane (about 1 cm^2^) in 1 mL of 0.1 M alcoholic ninhydrin solution contained in a tube. The tube was heated in boiling water for 3 min, and the solution diluted with 5 mL of water. The wettability of the membranes was investigated by the static water contact angle (SCA) measurement using the sessile drop method. A drop of water (5 µL) was injected using a micro-syringe with an automatic dispenser onto the membrane surface. The images were recorded using a CAM 200 device (KSV Instruments, Ltd., Helsinki, Finland). The measurements were repeated at least three times in different places in the membrane sample.

The surface zeta potential of the PVDF and PVDF/AL membranes was measured in a 2 mM KCl solution at pH 8.5 (Tris/HCl 20 mM) by using a Zetasizer Nano Series Nano ZS (Malvern instruments, Malvern, UK) equipped with a Zetasizer Nano cell kit. Polystyrene microspheres with a size of 202 ± 4 nm were used as the standard.

### 2.4. Activity Assays and Enzyme Stability Studies

The specific activity of free phosphotriesterase was evaluated at 25 °C using paraoxon-ethyl as a substrate. The hydrolysis of this substrate produces the release of 4-nitrophenol. The phosphotriesterase solution (10 µL, 0.1 g·L^−1^) was added to the substrate solution (1 mL) contained in a cuvette inside a spectrophotometer and the release of 4-nitrophenol was followed at 405 nm. In particular, the phosphotriesterase assay mixture consisted of a 1 mM paraoxon-ethyl solution buffered at pH 8.5 (Tris/HCl, 20 mM). The molar extinction coefficient of 4-nitrophenol estimated at pH 8.5 resulted in 15,837 M^−1^·cm^−1^. The phosphotriesterase specific activity represents the produced micromoles of 4-nitrophenol per minute per milligram of enzyme (µmol·min^−1^·mg_enz_^−1^). The specific activity was calculated using the following Equation (3):

(3) $$Specific\;activity=\frac{\frac{\Delta A}{min}\times V}{\varepsilon \times d\times E_{mass}}$$

In which ΔA is the increase in absorbance at 405 nm and ΔA/min represents the slope of the absorbance versus time plot, V (mL) is the volume of reaction, ε is the molar extinction coefficient of 4-nitrophenol, d (cm) represents the optical path of the cuvette, and E_mass_ (mg) is the amount of free or immobilized enzyme employed in the assay.

The specific activity of immobilized phosphotriesterase was measured by placing the enzyme loaded-membrane (area 4 cm^2^) in a batch reactor containing 5 mL of substrate solution. The formation of 4-nitrophenol was monitored, taking samples as a function of time.

The stability over time of both free and immobilized phosphotriesterase was estimated calculating half-life times, $t_{\frac{1}{2}}$ [25], by applying the following Equation (4):

(4) $$t_{\frac{1}{2}}=\frac{0.693}{K_{d}}$$

Here, *K_d_* (days^−1^) represents the deactivation constant of the phosphotriesterase:

(5) $$K_{d}=\frac{2.303}{t}log\frac{A_{E_{0}}}{A_{E_{t}}}$$

In this equation, t represents the operation time (days) while *A_E0_* and *A_Et_* indicate the initial phosphotriesterase-specific activity and the phosphotriesterase-specific activity at a certain operation time, respectively. 

### 2.5. Biocatalytic Membrane Reactor Set-Up

**Figure 1:**
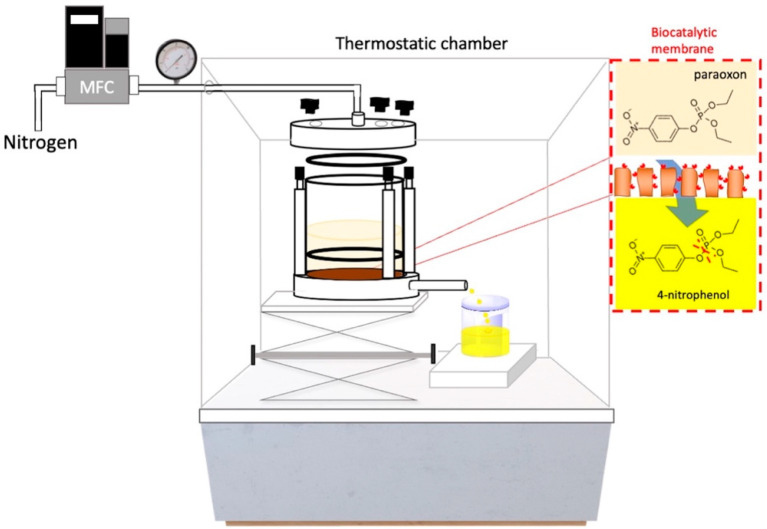
Schematic diagram of the biocatalytic membrane reactor system.

The experimental set-up used in this work is shown in Figure 1. The system consisted of a dead-end cell allocating the biocatalytic membrane, a mass flow controller (MFC, Brooks Instruments, Hatfield, PA, USA) to regulate the flow rate of nitrogen used to promote the driving force (ΔP) pressing the contaminated water through the membrane, a thermostatic chamber to regulate the reaction temperature (25 °C), and a pressure gauge (Wika, Milan, Italy).

The membrane void volume (*V_void_*) represented the BMR volume (mL) and was calculated as follows:

(6) $$V_{void}=\frac{{wt}_{w}-{wt}_{d}}{\rho_{s}}$$

where *wt_w_* is the membrane wet weight (g), *wt_d_* is the membrane dry weight (g), and *ρ_s_* is the density of the solution (approximated to the density of water: 1 g/mL).

The reactor was operated at a constant pressure of 40 mbar and a flow rate of 0.67 mL·min^−1^. The residence time (*τ*, min) was calculated as follows:

(7) $$\tau =\frac{V_{void}}{Flow\;rate}$$

The continuous BMR operated under time-invariant conditions and the concentration of the reaction product (4-nitrophenol) formed upon permeation of 1 mM paraoxon-ethyl solution through the membrane was measured in the volume fractions collected as a function of time at the outlet of the membrane, allowing us to calculate the enzyme activity and paraoxon degradation %.

### 2.6. Data Reproducibility

Experiments were conducted at least in triplicate on each individual sample, and the results were reported as the mean (±STD, standard deviation).

## 3. Results and Discussion

### 3.1. Screening of PVDF/AL Ratios on Enzyme-Loaded Membranes’ Performance

Membranes made of PVDF alone and blended with AL in different ratios (PVDF_90_/AL_10_, PVDF_75_/AL_25_ and PVDF_50_/AL_50_) were prepared. The morphological characterization by SEM analysis confirmed an asymmetric cross-sectional structure (Figure S1), as observed in [17].

**Figure 2:**
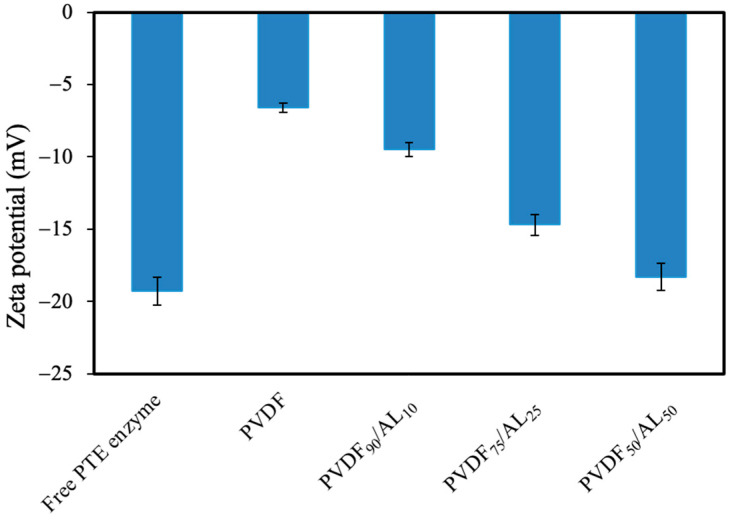
Surface zeta potential (mV) of free PTE, PVDF, PVDF_90_/AL_10_, PVDF_75_/AL_25_, and PVDF_50_/AL_50_ membranes measured in a 2 mM KCl solution at pH 8.5 (Tris/HCl 20 mM).

Before chemical functionalization, blank experiments were carried out, incubating the membranes in a phosphotriesterase solution of 0.1 g·L^−1^ (pH 8.5) for 2 h at 25 °C. The results showed that no enzyme adsorbed to the membranes. In the case of the PVDF membrane, this could be due to the limited interaction between the hydrophobic membrane and the enzyme dissolved in the aqueous buffer phase. The adsorption of phosphotriesterase on PVDF/AL membranes could be hindered by electrostatic repulsion between the enzyme and the membranes which are both negatively charged (Figure 2).

**Figure 3:**
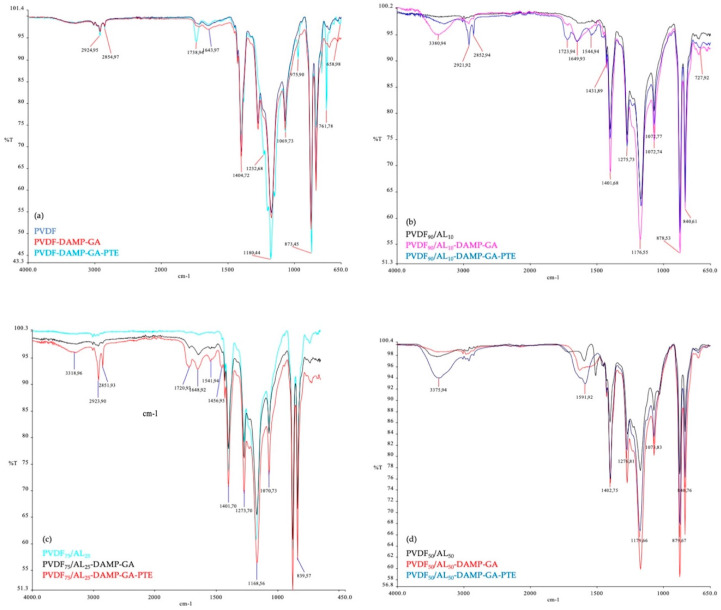
ATR-FTIR of PVDF (**a**), PVDF_90_/AL_10_ (**b**), PVDF_75_/AL_25_ (**c**), and PVDF_50_/AL_50_ (**d**) membranes.

To covalently immobilize the phosphotriesterase on the PVDF and PVDF/AL membranes, alkaline conditions and DAMP were used as described in Section 2. The PVDF polymer consists of –CH_2_CF_2_– groups; by treating it with DAMP at a high temperature (50 °C) and high pH (11), double bonds can be created (–CH=CF–) due to HF elimination followed by the grafting of amino groups, which subsequently reacted with glutaraldehyde [19]. The presence of free amino groups on both the PVDF and PVDF/AL membranes after treatment with DAMP was confirmed by the development of purple color in the ninhydrin test, and the quenching of the amino groups by glutaraldehyde was confirmed by the disappearance of the purple color in the ninhydrin test (as an example, Figure S2a–c shows the images of PVDF_75_/AL_25_, PVDF_75_/AL_25_-DAMP, and PVDF_75_/AL_25_-DAMP-GA membranes). ATR-FTIR (Figure 3) analyses were also carried out to confirm the presence of the chemical groups at each reaction step.

Glutaraldehyde is a crosslinker that enables the immobilization of biomolecules under mild reaction conditions by the formation of Schiff bases between the carbonyl group and the ε-amino groups of lysine on the surfaces of the biomolecules [26]. The chemically functionalized membranes were then incubated with the enzyme solution (0.1 mg·mL^−1^, pH 8.5, 25 °C). As illustrated in Table 1, the amount of immobilized enzyme, calculated by the mass balance according to Equation (1), is of the same order of magnitude for the different membranes. The results of the specific activity show that the immobilization of the enzyme on the PVDF and PVDF/AL membranes leads to a decrease in phosphotriesterase-specific activity compared to the free enzyme (Table 1). The decrease in the specific activity of the enzyme after immobilization on insoluble supports is frequently reported in the literature and is ascribed to crowding phenomena, diffusion limitation, and enzyme stiffening [27].

The immobilized phosphotriesterase showed the highest specific activity when immobilized on membranes containing 10% or 25% of AL. In fact, the high hydrophilicity of PVDF_75_/AL_25_ (WCA = 89 ± 3°), which is due to the presence of AL, makes the blended membrane better wetted by the aqueous medium in which the pesticide (paraoxon-ethyl) is dissolved, compared to the PVDF membrane (WCA = 115 ± 2°). Unexpectedly, a further increase in the percentage concentrations of AL from 25% to 50% in the PVDF_50_/AL_50_ (the water contact angle could not be measured as the droplet was adsorbed immediately [17]) caused a decrease in the specific activity of the immobilized phosphotriesterase. One factor contributing to this result could be the membrane charge. In fact, as shown in Figure 2, the PVDF_50_/AL_50_ membrane has the most negative surface zeta potential. 

**Figure 4:**
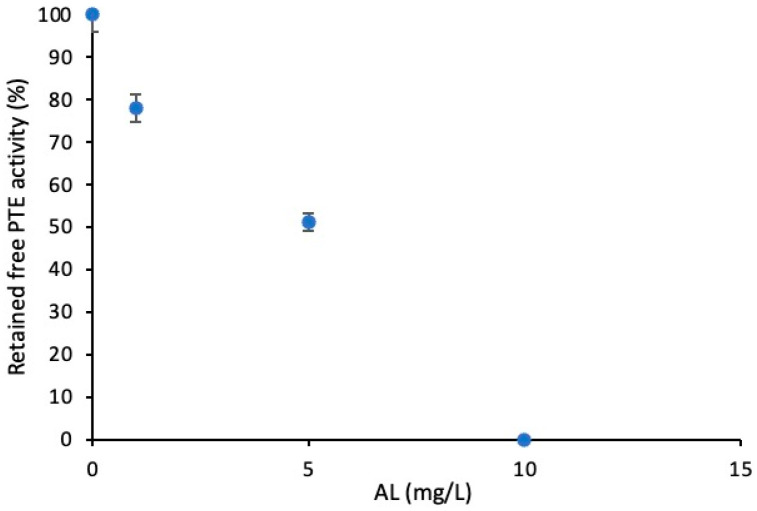
Free PTE activity as a function of AL concentration (mg/L).

The surface charge of immobilized enzyme carriers is one of the most important factors determining the catalytic properties of enzymes [28]. Various studies in the literature have shown that the surface charge of the carrier can influence both the configuration and the interaction with the substrate of an immobilized enzyme [29,30,31]. However, in our case, the surface zeta potential decreased only slightly (from −14.7 to −18.3 mV) for PVDF_75_/AL_25_ and PVDF_50_/AL_50_. Thus, these results indicate that the surface charge of PVDF_50_/AL_50_ is not the main reason for the decreased specific activity of phosphotriesterase. The decrease in phosphotriesterase activity for high AL content could also be due to an inhibitory effect of this polymer. The inhibition effect of lignin on enzymes such as cellulase and tyrosinase has been described in the literature [32,33]. In particular, the inhibition can be ascribed to the polyphenolic structure of lignin [15]; moreover, we have already reported in our previous work that polyphenols like hydroxytyrosol act as a competitive phosphotriesterase inhibitor [34]. Therefore, free phosphotriesterase was tested in an aqueous solution with AL at increasing concentrations to better understand the effects on the enzyme activity. It was observed that the enzyme activity decreased linearly with AL concentration (Figure 4).

**Figure 5:**
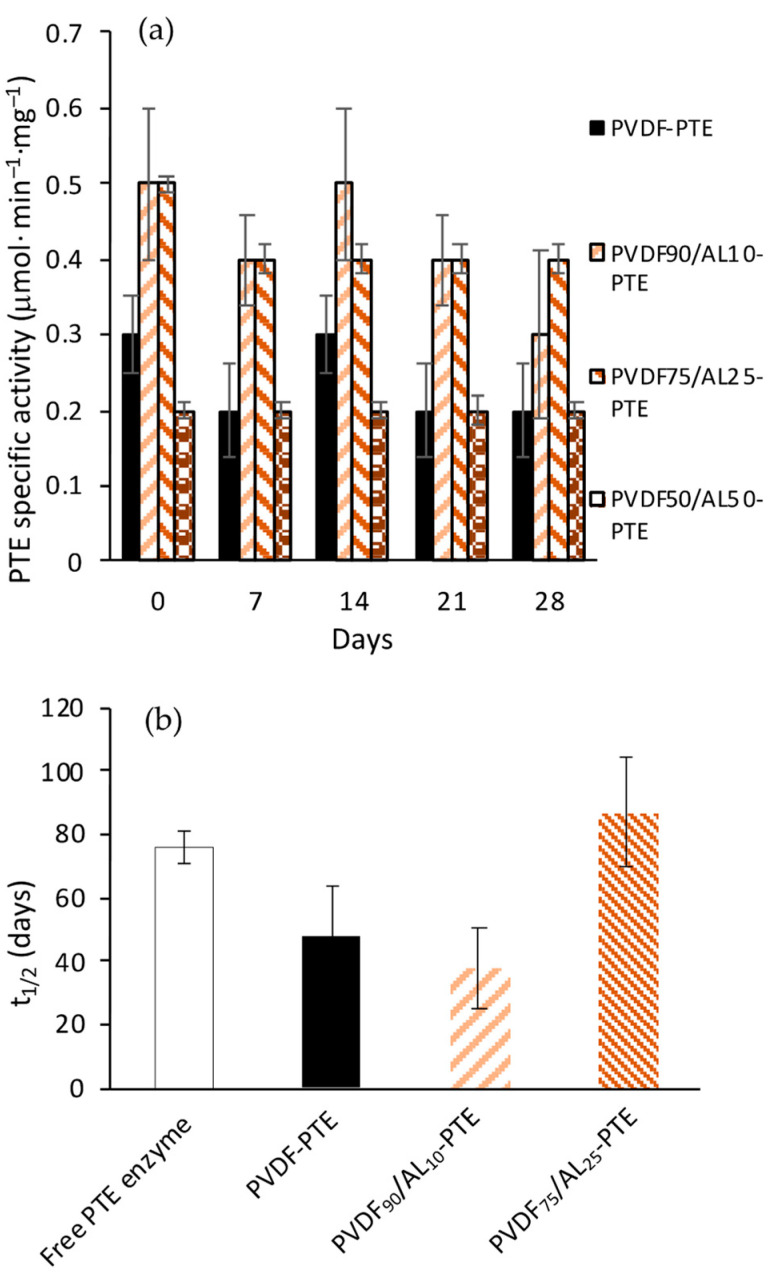
Stability of immobilized PTE over time (**a**). Half-life time (t_1/2_) of free and immobilized PTE (**b**).

To summarize, it seems that the presence of AL in the PVDF membrane shows a double effect: beneficial on the enzyme activity of the immobilized enzyme up to a certain concentration (up to 25%) and detrimental at higher concentrations. The specific activity of enzyme-loaded membranes (Figure 5a) and free enzymes (Figure S3) as a function of time shows that the enzyme immobilized on PVDF_75_/AL_25_ has the highest half-life time (Figure 5b). Considering these outcomes, the membrane containing 25% of AL (PVDF_75_/AL_25_) was selected to develop the BMR. It is worth noting that, despite the significant reduction in enzyme-specific activity compared to the free enzyme, the overall enzyme-loaded membrane retains sufficient catalytic activity and can perform the degradation of paraoxon-ethyl in a continuous process when operating as a BMR.

### 3.2. Paraoxon-Ethyl Degradation by BMR

The enzymatic degradation of paraoxon-ethyl was carried out by assembling the PVDF_75_/AL_25_ biocatalytic membrane in the cell depicted in Figure 1. To maximize the degree of paraoxon-ethyl degradation, three PVDF_75_/AL_25_ membranes with different void volumes (i.e., reactor volume) were prepared. The membranes with different void volumes were obtained by increasing the membrane wall thickness (Table 2). As expected, the pure water permeance decreased as an effect of the increased membrane thickness.

**Figure 6:**
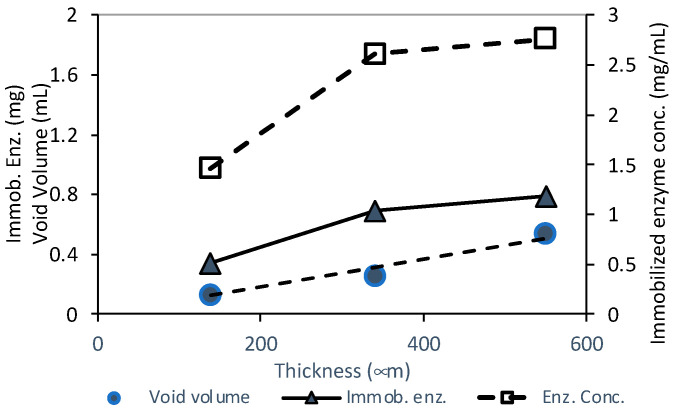
Behavior of void (reactor) volume, enzyme amount, and enzyme concentration in BMR with membranes of different thicknesses.

Figure 6 illustrates the behavior of the reactors’ volume, immobilized enzyme amount, and concentration as a function of membrane thickness. Although the reactor volume increased with the membrane thickness, the amount of immobilized enzyme and its concentration leveled-off at the highest thickness/void volume. This means that the protocol for immobilizing the enzyme must be adapted in order to increase the amount of enzyme and keep the concentration constant. 

**Figure 7:**
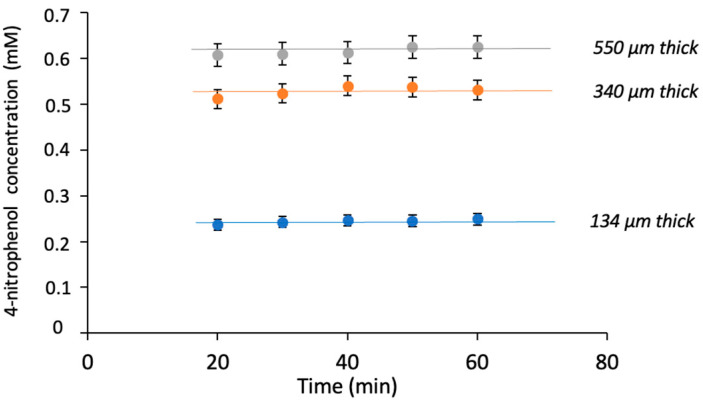
Concentration of 4-nitrophenol coming from BMR sampled over time for membranes with different thicknesses (i.e., different void volumes).

The behavior of the concentration of the reaction product at the outlet of the individual BMRs as a function of time (Figure 7) confirmed that the BMRs work under time-invariant conditions and can be approximated to a continuous tank reactor system.

**Figure 8:**
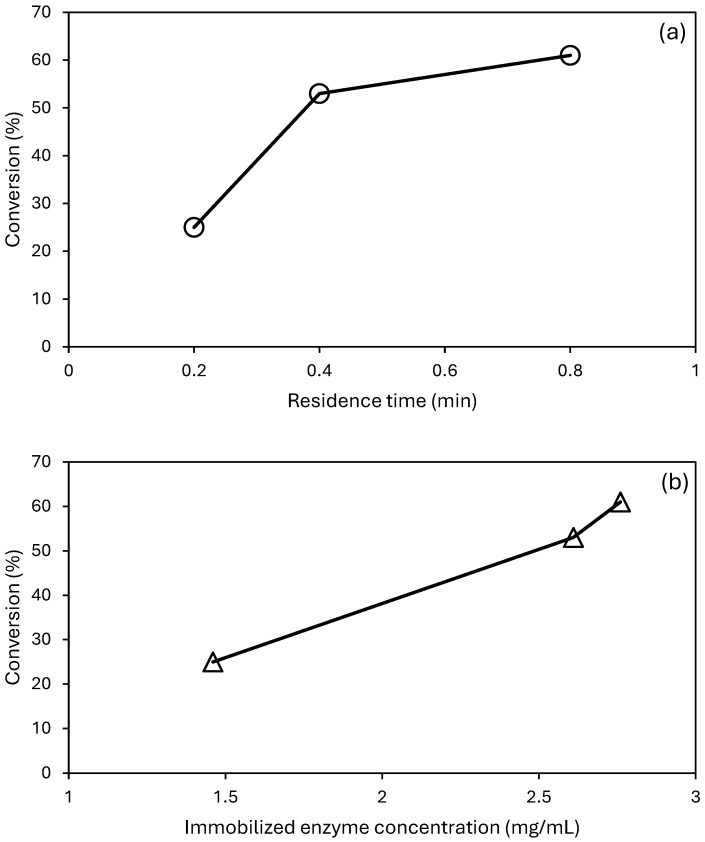
Percentage of paraoxon-ethyl degradation as a function of residence time (**a**) and immobilized PTE concentration (**b**).

The flow rate through the biocatalytic membranes (0.67 mL·min^−1^) was kept constant for all the membranes and was obtained by applying a pressure of 40 mbar. This allowed us to change the residence time of the reagent within each BMR without changing the fluid dynamics. In Figure 8, the percentage of paraoxon-ethyl degradation as a function of residence time (a) and immobilized PTE amount (b) is reported. 

As can be seen, the paraoxon-ethyl degradation increased with the enzyme concentration (Figure 8b), while it approached a plateau as a function of residence time (Figure 8a). This behavior suggests that a further enzyme concentration increase is necessary to benefit from the higher residence time and reach a higher percentage of paraoxon degradation. Previous studies have shown that the performance of PTE-loaded membranes is influenced by several parameters, including the amount of immobilized enzyme, membrane wettability, and residence time [34]. When considering the cases under similar conditions of immobilized enzyme concentration and membrane wettability, the results obtained in the present work show better performance. For example, PTE immobilized on a hydrophilic membrane of regenerated cellulose (concentration of immobilized enzyme = 1.6 mg·mL^−1^) showed a specific activity of about 0.25 µmol·min^−1^·mg^−1^ [34], while PTE immobilized on hydrophilized PVDF_75_/AL_25_ (concentration of immobilized enzyme = 1.5 mg·mL^−1^) showed a specific activity of 0.5 µmol·min^−1^·mg^−1^.

## 4. Conclusions

The present research focused on the preparation of hydrophobic PVDF and hydrophilized PVDF/AL membranes to prove that immobilized enzymes are strongly affected by the hydrophobic microenvironment. The biocatalytic membranes were used for the degradation of an organophosphate pesticide (paraoxon-ethyl) contained in aqueous streams. The influence of the PVDF/AL mass ratio on the enzyme activity was investigated. The immobilized phosphotriesterase showed the highest activity (0.5 µmol·min^−1^·mg^−1^) in a PVDF membrane with 25% AL. The specific activity was about 50% higher than that of phosphotriesterase immobilized on a pure PVDF hydrophobic membrane. The degradation efficiency of the pesticide was studied in a biocatalytic membrane reactor (BMR) as a function of residence time. Although the BMR needs further optimization, the work showed that adjusting the membrane void volume enables an increase in the residence time and thus the degradation of the pesticide. The system appears to be promising for applications in areas such as bioremediation and biorecognition.

## Figures and Tables

**Table 1:** Amounts of immobilized enzyme and specific activity related to glutaraldehyde functionalized membranes.

Free Enzyme/Membrane	Free Enzyme (mg/mL)	Immob. Enzyme (mg_E_·g_mem_^−1^)	Specific Activity (µmol·min^−1^·mg^−1^)
Free enzyme	9.9 × 10^−4^	-	3.6 ± 0.3
PVDF-DAMP-GA	-	6.2 (±0.4)	0.3 (±0.05)
PVDF_90_/AL_10_-DAMP-GA	-	7.5 (±0.6)	0.5 (±0.1)
PVDF_75_/AL_25_-DAMP-GA	-	6.5 (±0.3)	0.5 (±0.01)
PVDF_50_/AL_50_-DAMP-GA	-	8.5 (±0.5)	0.2 (±0.01)

**Table 2:** Porosity, pure water permeance, and void volume related to PVDF_75_/AL_25_-DAMP-GA membranes of different thicknesses.

Membrane Type	Thickness (µm)	Porosity (%)	Pure Water Permeance (L·h^−1^·m^−2^·bar^−1^)	Void Volume (mL)
PVDF_75_/AL_25_-DAMP-GA	138 (±2) *	80	577 (±26) *	0.13
	340 (±3)	66	560 (±30)	0.25
	550 (±7)	79	400 (±40)	0.54

* Data reproduced from a previous work [17].

## Data Availability

The raw data presented in this study are available on request from the corresponding author due to a period of confidentiality required within the project in progress.

## Supplementary Materials

The following supporting information can be downloaded at: https://www.mdpi.com/article/10.3390/membranes14090186/s1, Figure S1: Cross-section SEM images of PVDF (a), PVDF_90_/AL_10_ (b) membranes; Figure S2: Images of PVDF_75_/AL_25_ (a), PVDF_75_/AL_25_-DAMP (b) and PVDF_75_/AL_25_-DAMP-GA (c) membranes after ninhydrin test; Figure S3: Specific activity of free enzyme as a function of time.
