# Supplementary material for: Tuning the Properties of Polyvinylidene Fluoride/Alkali Lignin Membranes to Develop a Biocatalytic Membrane Reactor for an Organophosphorus Pesticide Degradation
Source: Membranes (Basel). 2024 Aug 28;14(9):186. doi: 10.3390/membranes14090186 (PMC11434455; doi:10.3390/membranes14090186)
Supplement: Supplementary file 1 [file membranes-14-00186-s001.zip › membranes-3166551-supplementary.pdf]

Supplementary Materials

Tuning the Properties of Polyvinylidene Fluoride/Alkali Lignin Membranes to Develop a Biocatalytic Membrane Reactor for an Organophosphorus Pesticide Degradation

Serena Regina, Giuseppe Vitola, Rosalinda Mazzei and Lidietta Giorno *

National Research Council of Italy, Institute on Membrane Technology, CNR-ITM, 87036 Rende, Italy
* Correspondence: l.giorno@itm.cnr.it; Tel.: +39-0984492050

Cross sections

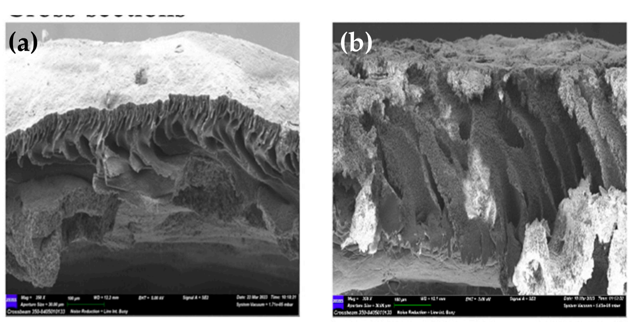

Figure S1. Cross-section SEM images of PVDF (**a**), $PVDF_{90}/AL_{10}$ (**b**) membranes.

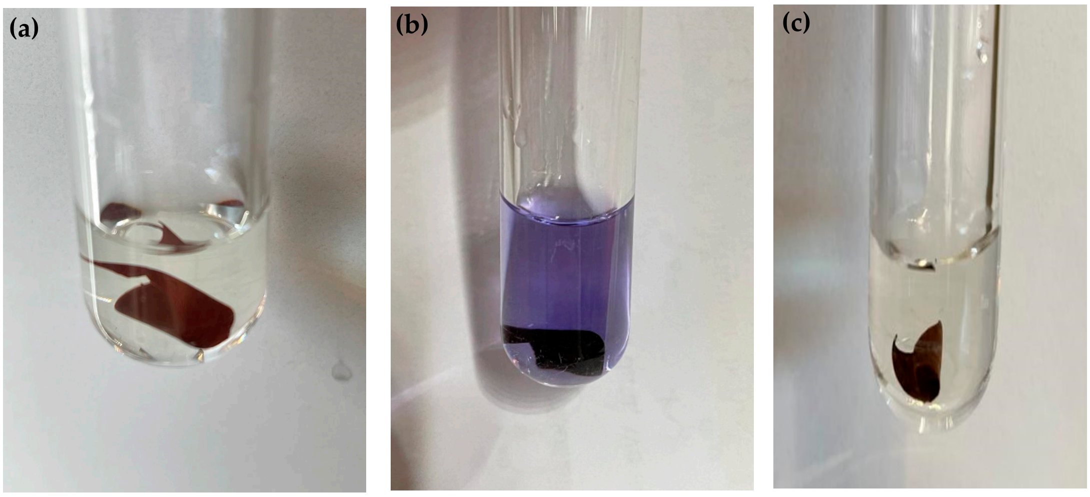

Figure S2. Images of $PVDF_{75}/AL_{25}$ (**a**), $PVDF_{75}/AL_{25}$-DAMP (**b**) and $PVDF_{75}/AL_{25}$-DAMP-GA (**c**) membranes after ninhydrin test.

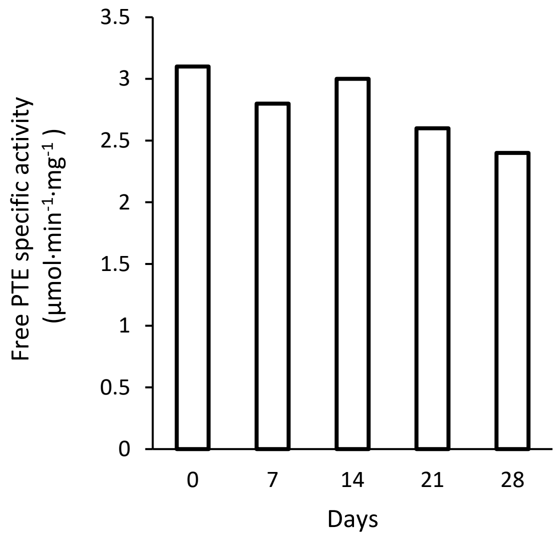

Figure S3. Specific activity of free enzyme as a function of time.
